# Targeting Soluble Guanylyl Cyclase during Ischemia and Reperfusion

**DOI:** 10.3390/cells12141903

**Published:** 2023-07-21

**Authors:** Eric H. Mace, Melissa J. Kimlinger, Frederic T. Billings, Marcos G. Lopez

**Affiliations:** 1 Department of Surgery, Vanderbilt University Medical Center, Medical Center North, Suite CCC-4312, 1161 21st Avenue South, Nashville, TN 37232-2730, USA; 2 Vanderbilt University School of Medicine, 428 Eskind Family Biomedical Library and Learning Center, Nashville, TN 37240-0002, USA; 3 Department of Anesthesiology, Division of Critical Care Medicine, Vanderbilt University Medical Center, Medical Arts Building, Suite 422, 1211 21st Avenue South, Nashville, TN 37212-1750, USA

**Keywords:** soluble guanylyl cyclase, sGC, ischemia reperfusion injury, sGC stimulator, sGC activator, vasodilation, oxidative injury, cardioprotection, neuroprotection

## Abstract

Ischemia and reperfusion (IR) damage organs and contribute to many disease states. Few effective treatments exist that attenuate IR injury. The augmentation of nitric oxide (NO) signaling remains a promising therapeutic target for IR injury. NO binds to soluble guanylyl cyclase (sGC) to regulate vasodilation, maintain endothelial barrier integrity, and modulate inflammation through the production of cyclic-GMP in vascular smooth muscle. Pharmacologic sGC stimulators and activators have recently been developed. In preclinical studies, sGC stimulators, which augment the reduced form of sGC, and activators, which activate the oxidized non-NO binding form of sGC, increase vasodilation and decrease cardiac, cerebral, renal, pulmonary, and hepatic injury following IR. These effects may be a result of the improved regulation of perfusion and decreased oxidative injury during IR. sGC stimulators are now used clinically to treat some chronic conditions such as heart failure and pulmonary hypertension. Clinical trials of sGC activators have been terminated secondary to adverse side effects including hypotension. Additional clinical studies to investigate the effects of sGC stimulation and activation during acute conditions, such as IR, are warranted.

## 1. Introduction

Ischemia and reperfusion (IR) injury contributes to many pathophysiological processes including stroke, hemorrhage, and trauma, and affects organ function and recovery following organ transplantation, coronary revascularization, and thrombolytic therapy for peripheral vascular disease. Despite the longstanding effort to develop pharmacologic therapies to ameliorate the damage caused by IR, there remain few impactful interventions. The pathophysiology of IR is broad, and treatment strategies frequently aim to reduce the acute oxidative and inflammatory response [1,2,3]. Due to the importance of blood flow and the vascular response to ischemia and reperfusion, new treatments targeting the vasodilatory alterations that occur after IR have recently been investigated. Interventions aimed at augmenting nitric oxide signaling and the regulation of vascular responsiveness have been a key focus of many pre-clinical and clinical studies [4,5,6]. Soluble guanylyl cyclase (sGC) regulates vascular function in the nitric oxide signaling pathway. sGC-specific stimulators and activators target this enzyme to affect vascular tone and perfusion [7,8,9]. The modulation of sGC function during acute periods of IR provides an exciting opportunity to develop therapies that decrease IR injury. Herein, we review the regulation of vasodilation with a focus on nitric oxide signaling and sGC function, vascular dysfunction during IR, and efforts to target IR using sGC stimulators and activators in preclinical and clinical studies.

## 2. Regulation of Vasodilation

The vascular endothelium regulates hemostasis, immune cell trafficking, vascular permeability, and angiogenesis. Perhaps most importantly, the endothelium regulates vascular tone to control tissue perfusion [10,11]. A substance termed “endothelium-derived relaxing factor” (EDRF) increased the production of the second messenger cyclic 3′,5′-guanosine monophosphate (cGMP) and was posited as the mechanism by which the endothelium mediates vasodilation. EDRF was subsequently identified as nitric oxide (NO) [12,13]. While several other mediators, such as prostaglandins, adenosine, ATP-sensitive potassium channel activators, and endothelium-derived hyperpolarizing factors, impact vascular tone, endothelium-dependent vasodilation is in large part a function of NO [14,15,16,17].

### 2.1. Nitric Oxide Synthesis and Function

NO-synthase I (neuronal or nNOS), NO-synthase II (inducible or iNOS), and NO-synthase III (endothelial or eNOS) produce NO from L-arginine [18]. These enzymes are widely expressed. For example, nNOS has been detected not only in nerves but also in endothelium and smooth myocytes, while iNOS has been documented in all nucleated cells of the cardiovascular system [19,20,21]. eNOS is present in cardiac myocytes and platelets, and it is the predominant isoform in endothelial cells. All of these enzymes contain a reductase and an oxygenase domain, and they all require multiple cofactors to generate NO [22]. The eNOS conversion of L-arginine to NO requires two independent steps; the first is NADPH-dependent and the second utilizes O_2_ and NADPH to generate NO and L-citrulline. These steps are accelerated by the cofactor tetrahydrobiopterin, which also facilitates the obligatory dimerization of NOS [23,24].

**Figure 1:**
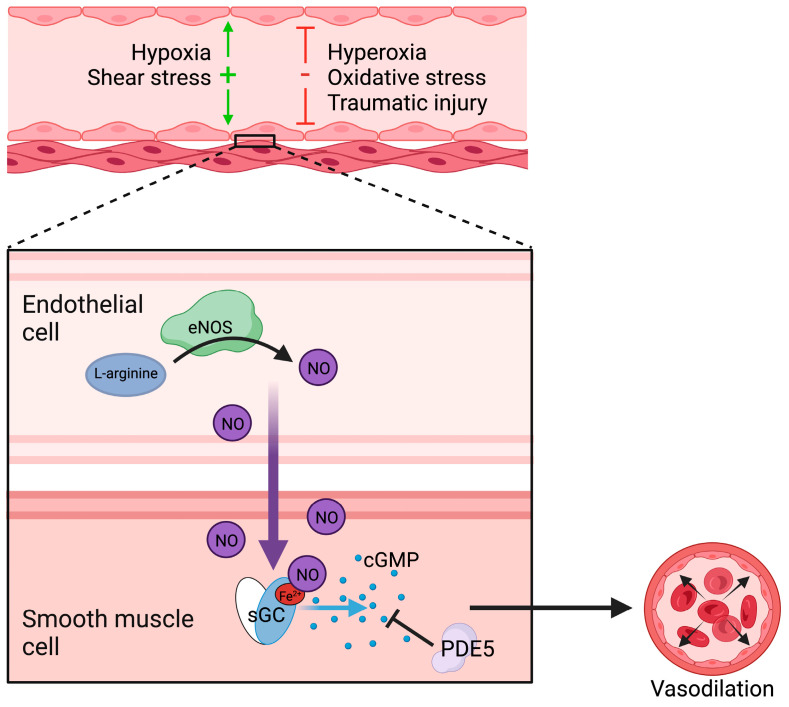
Regulation of vasodilation by nitric oxide and soluble guanylyl cyclase. Nitric oxide (NO) is generated in the endothelium, diffuses into smooth muscle, binds to soluble guanylyl cyclase (sGC), where it induces a conformational change that catalyzes the generation cyclic guanosine monophosphate (cGMP). cGMP activates subsequent kinases resulting in vasodilation. Phosphodiesterase 5 (PDE–5) metabolizes cGMP. Hypoxia and shear stress stimulate endothelium-mediated vasodilation, whereas hyperoxia, oxidative damage, and trauma impair NO–mediated vasodilation.

eNOS activity is controlled by transcriptional and post-translational regulations. eNOS transcription is enhanced by shear stress, hydrogen peroxide, oxidized LDL, and hypoxia, while eNOS mRNA stability is affected by these factors and others, such as the presence of lipopolysaccharide [25]. eNOS function is, furthermore, dependent upon calcium binding to calmodulin for activation [26]. Each of these factors affects eNOS production of NO, and NO itself is highly reactive. Specifically, NO reacts with superoxide to form peroxynitrite or is inactivated by conversion into nitrate and oxidation of hemoglobin [27,28]. NO readily diffuses across cell membranes and has protective, regulatory, and sometimes deleterious effects which are mediated by direct interaction with metals, lipid peroxides, and other radicals, such as superoxide and the hydroxyl radical [29]. NO-induced vasodilation, as well as its inhibitory effects on platelet adhesion and aggregation, occurs via NO binding to sGC in the cytoplasm of smooth myocytes inducing a conformational change in sGC that promotes the generation of cGMP (Figure 1) [30,31].

### 2.2. Soluble Guanylyl Cyclase Regulation and Function

Guanylyl cyclase is a heterodimer heme-protein with an α-subunit and a β-subunit. The β-subunit contains a ferrous heme group that catalyzes the conversion of guanosine-5′-triphosphate (GTP) into cGMP and pyrophosphate. It exists in both membrane-bound and free, cytosolic forms, the latter of which binds NO. sGC is an allosterically activated enzyme composed of a sensor module, a transducer module, and a catalytic module. Binding of NO to the sGC sensor module, which relies upon the presence of a ferrous heme (Fe^2+^) moiety present in the β-subunit, induces a conformational change that is conveyed to the transducer module and subsequently allosterically transmitted to the catalytic module to open the GTP binding cleft and generate cGMP [7,30,31,32]. The oxidation of the sGC heme moiety (Fe^2+^ → Fe^3+^) may result from IR-induced oxidative stress. sGC heme oxidation or dissociation of the heme moiety eliminates its ability to bind NO and renders the enzyme inactive [31]. The product of sGC, cGMP, activates multiple cGMP-dependent protein kinases such as protein kinase G (PKG) in vascular smooth myocytes, platelets, and the endothelium. The net effect of this cGMP signal transduction is vascular relaxation via decreased intracellular calcium in vascular smooth myocytes and the decreased calcium sensitivity of contractile proteins [33,34]. In addition to its vascular effects, sGC activation decreases platelet activation, aggregation, secretion, and adhesion, impacting coagulation [35]. Each of these functions may be affected by IR-induced changes in sGC activity and could exacerbate IR-related injury.

## 3. Vascular Dysfunction in Ischemia and Reperfusion

Organs are variably susceptible to IR injury. For example, the brain, heart, and kidney are at particularly high risk of IR-related injury. In addition to local injury, IR induces systemic effects that lead to inflammation, vascular dysfunction, and diffuse organ dysfunction [36,37]. Vascular dysfunction may mediate the systemic effects of IR injury.

### 3.1. Endothelial Responses to Ischemia-Reperfusion

IR injury leads to endothelial injury and dysfunction. Cell swelling is uniformly observed among endothelial cells after IR injury and may be the result of oxidative damage to cellular membranes, ion dysregulation, and osmotic stress [38]. Membrane depolarization occurs early after IR due to the failure of ATP-dependent ion exchange, but it may also result from alterations in K^+^ channels in oxidizing conditions [39]. Additionally, oxidants such as hydrogen peroxide and superoxide induce endothelial cell apoptosis through caspase pathways [40], and these effects are further aggravated following reperfusion. In cardiac tissue, for example, microvascular injury as marked by endothelial nuclear damage, low cell junction density, and microsphere extravasation was more severe after ischemia and one hour of reperfusion than after ischemia alone [41]. Effects at the cellular level likely contribute to systemic endothelial dysfunction induced by IR.

Endothelial dysfunction after IR is categorized by decreases in vasodilatory capacity and leukocyte recruitment, and the NO-sGC-cGMP signaling pathway is central to these alterations. Basal and agonist-mediated NO production is decreased following reperfusion, and this decrease persists for hours, possibly due to superoxide quenching of NO [42,43,44]. eNOS uncoupling may be induced by the consumption of L-arginine and tetrahydrobiopterin by superoxide and hydrogen peroxide. Uncoupled eNOS produces the free radical peroxynitrite rather than NO, further impairing endothelial function [45,46]. In this setting, the β-subunit heme iron of sGC may be oxidized, preventing the binding of NO and production of cGMP [47]. In addition to oxidative injury, alterations to cell adhesion molecules such as selectins, ß2 integrins, and immunoglobulins lead to increased immune cell adherence to the damaged endothelium and increased inflammation, exacerbating reperfusion injury [48,49]. On the whole, a dysfunctional endothelium leads to increased organ damage during IR.

### 3.2. Impact of Endothelial Dysfunction on Organ Injury

Endothelial dysfunction is a systemic pathology that affects large arteries, resistance vessels, and the microcirculation [50]. It is associated with organ injury in pre-clinical and clinical settings. The assessment of reactive hyperemia, the increase in blood flow following a brief period of ischemia, is a useful method to quantify endothelial function in clinical studies because the magnitude of increased blood flow is an endothelium-mediated response. Flow-mediated dilation (FMD) and fingertip pulse amplitude tonometry are two non-invasive techniques used to measure reactive hyperemia, and increased FMD and pulse amplitude tonometry responses are associated with cardiovascular disease risk and decreased renal function [51,52,53,54,55].

The endothelium plays a critical role in the development of atherosclerosis and atherosclerosis-related thrombus formation [56]. There is a strong correlation between brachial artery FMD and coronary artery endothelium-mediated vasodilation in response to exogenous acetylcholine [57,58]. Chronic endothelial dysfunction measured by FMD is an accurate predictor of future cardiovascular events such as myocardial infarction, stroke, coronary disease, and claudication [59]. In a preclinical model of myocardial ischemia with isolated rat hearts, impairment of endothelium-dependent vasodilation occurred early in the ischemic period and preceded myocardial dysfunction [60]. The interaction of oxygen and the endothelium may also be important in myocardial perfusion. In swine with induced coronary artery stenosis, for example, the administration of hyperoxia decreased coronary blood flow and led to myocardial ischemia and decreased cardiac output [61]. These changes may be mediated by endothelium-dependent vasoconstriction in response to hyperoxia [62]. Hyperoxia promotes increased oxidative stress, and oxidative stress may further contribute to hyperoxic vasoconstriction [63,64,65]. In this way, the endothelium plays a critical role in the regulation of perfusion, and endothelial dysfunction may result in dysregulated perfusion and may contribute to the development of cardiovascular disease. The effects of endothelial dysfunction, however, are not isolated to the cardiovascular system.

Cerebral dysfunction has been linked to a dysfunctional endothelium. In patients with shock or respiratory failure, a lower reactive hyperemia index measured with digital pulse amplitude tonometry was associated with increased incidence and longer duration of delirium [66]. Increased endothelial activation and blood-brain barrier injury was associated with fewer delirium-free days in critically ill patients with organ failure [67]. Increased systemic oxidative stress has also been associated with delirium in patients undergoing cardiac surgery [68]. Studies are ongoing to determine if this is related to the effects of oxidative stress on endothelial function [69]. The endothelium is a critical regulator of cerebral blood flow, and injury to endothelial cells is a contributing factor to stroke and cerebrovascular disease [70]. Clinically, impaired brachial artery FMD at the time of ischemic stroke has been associated with worse long term neurologic outcomes [71]. The effects of oxygen on the degree of injury in stroke may, in part, depend on endothelial function. In a murine model of middle cerebral artery occlusion, hyperoxia decreased infarct size and increased perfusion of the ischemic brain tissue in wild type mice [72]. In eNOS knockout mice in this study, however, hyperoxia reduced perfusion and increased infarct size. These results suggest that eNOS and intact NO signaling protect the brain following IR.

In addition to the endothelium’s role in regulating blood flow, other endothelial functions may be important to brain dysfunction and injury in IR settings. For example, the endothelium regulates inflammation and leukocyte-trafficking which influence cerebral injury in acute ischemic stroke [73]. The presence of intact NO-sGC signaling may promote an intact endothelial barrier by increasing phosphorylated vasodilator-stimulated phosphoprotein (VASP). VASP supports the integrity of tight- and adherence-junctions and promotes actin polymerization and microtubular assembly. These processes maintain the cytoskeleton and endothelial barrier [74]. Leukocytes may exacerbate injury by releasing additional free radicals, proteases, and inflammatory cytokines [75]. Natalizumab, a monoclonal antibody targeting α-4 integrin, decreases leukocyte trafficking across endothelial cells and has been investigated as a potential treatment to reduce injury in this setting. In randomized trials, however, natalizumab failed to reduce infarct size or functional outcomes after ischemic stroke [76,77]. Additional investigation in the modulation of inflammation, endothelial barrier function, and leukocyte trafficking is warranted as a means to reduce IR-related injury.

The prevalence of endothelial dysfunction during critical illness and its association with end-organ injury establish the endothelium as a promising target for pharmacologic intervention. Endothelium-generated NO is critical to normal endothelial function, and NO bioavailability or binding to sGC may be impaired during IR. Therefore, the NO-sGC-cGMP pathway is an important target of therapies aimed at reducing IR injury.

## 4. Targeting Soluble Guanylyl Cyclase in Ischemia-Reperfusion

Due to the large potential clinical impact of reperfusion injury, numerous therapies have been investigated to prevent and decrease IR injury [1,78,79,80,81]. In vitro treatments that reduce oxidative stress improve endothelial function, but these therapies have not translated to clinical utility [1,78]. In contrast, treatments that act directly in the NO signaling pathway have demonstrated utility in IR settings. For example, components of the NO signaling pathway, such as the NO-precursor L-arginine and direct NO donors, reduce myocardial infarct size and improve myocardial function in both in vivo and in vitro models [79,80,81,82]. NO-donating drugs are commonly used in clinical settings. For example, organic nitrates treat ischemic pain in cardiovascular disease, and gaseous NO treats pulmonary hypertension. NO donors also have therapeutic limitations. The long-term use of nitrates leads to tolerance, possibly through the oxidation of the heme moiety on the β-subunit of sGC [83]. Additionally, NO may have its own cytotoxic effects by reacting with superoxide to produce peroxynitrite, as well as through the formation of cyanide and methemoglobin from nitroprusside [23,84,85].

NO mediates some of its beneficial effects through the sGC-cGMP pathway. sGC is an attractive therapeutic target due to the potential to amplify cGMP signaling while avoiding NO-related potential side effects [86]. Phosphodiesterase-5 (PDE-5) inhibitors, including sildenafil, tadalafil, and vardenafil, inhibit the breakdown of cGMP. In pre-clinical studies, these drugs attenuate cardiac IR injury, whereas nitrates have not [87]. However, while initial studies examining the effects of PDE-5 inhibition to enhance cGMP signaling showed a beneficial effect of sildenafil in heart failure patients, subsequent clinical trials have shown little or no cardioprotective effect [88,89,90]. In patients undergoing cardiac surgery, a setting where IR injury is common, intravenous sildenafil did not decrease renal injury and in fact may cause harm [91]. While phosphodiesterase inhibitors have limited benefit in reducing organ injury, drugs that directly modulate sGC activity are currently under investigation [92].

### Soluble Guanylyl Cyclase Stimulators and Activators

**Figure 2:**
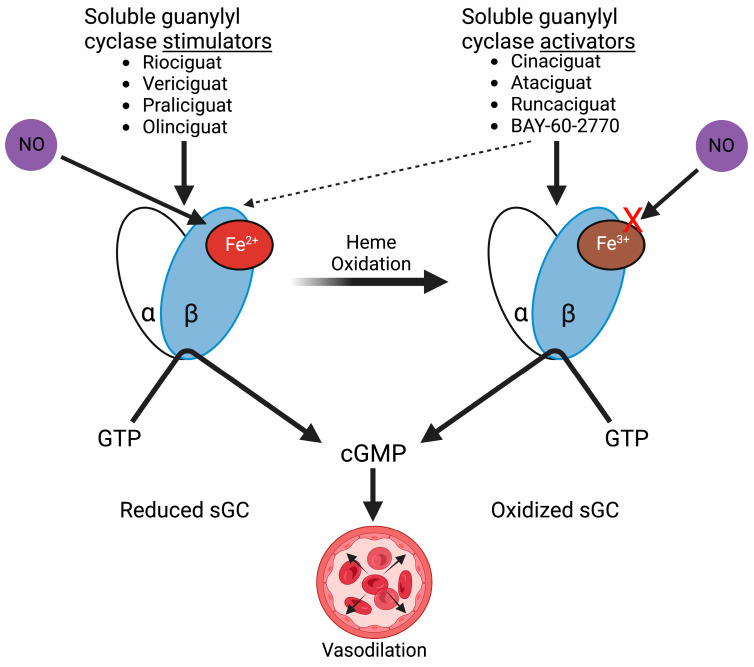
Soluble guanylyl cyclase (sGC) stimulators and activators. sGC stimulators and nitric oxide (NO) bind to reduced heme (Fe^2+^) sGC, whereas sGC activators preferentially bind and activate oxidized (Fe^3+^) sGC. sGC activators can also activate the reduced heme enzyme but to a lesser extent. sGC catalyzes the production of cyclic guanosine monophosphate (cGMP) from guanosine triphosphate (GTP), resulting in vasodilation.

sGC stimulators and activators promote NO signaling by directly binding to and activating sGC, increasing the production of cGMP. Stimulators differ from activators due to their dependence on the oxidation state of the heme binding site on the β subunit of the enzyme (Figure 2). sGC stimulators require the presence of ferrous, reduced heme to bind to and activate sGC. sGC stimulators have a strong synergistic effect with NO and thereby sensitize sGC to endogenously produce NO [31,93]. YC-1 was the first synthesized sGC stimulator and was noted to inhibit platelet aggregation and ATP release in a concentration-dependent manner [9]. High-throughput screening led to the development of multiple sGC stimulators with improved potency and specificity for sGC [7,8]. Riociguat, for example, increases the activity of sGC in vitro up to 73-fold and works synergistically with NO to increase sGC activity up to 122-fold [92]. While sGC stimulators are limited to acting upon reduced, ferrous sGC, sGC activators effectively and preferentially bind and activate oxidized, ferric sGC and also heme-free apo-sGC. sGC activators activate sGC irrespective of NO binding. They produce an additive rather than a synergistic effect with NO-binding [31]. sGC activators furthermore reduce the proteasomal degradation of apo-sGC after oxidation-induced damage [92]. With the increased availability of molecules directly targeting sGC, research has focused on the role of these drugs in improving the response to IR in several organ injury models.

## 5. Protective Effects of sGC Stimulation and Activation

The direct pharmacologic stimulation of sGC may be beneficial in many disease states due to the broad pathophysiological impact of endothelial dysfunction. sGC stimulators and activators have been studied for their protective effects on cardiac, cerebral, renal, pulmonary, and vascular tissue due to the consequences of IR on these tissues. Pre-clinical studies of sGC stimulators and activators are summarized in Table 1.

### 5.1. Cardiac Protection

In cardiovascular disease, sGC stimulators and activators have been extensively studied for their effects on vascular function and ischemic injuries. Cinaciguat, an sGC activator, has cardioprotective effects in a model of global cardiac ischemia [94]. In this rat model of myocardial infarction, oral cinaciguat treatment decreased infarct size, improved cardiac performance, reduced oxidative stress, and reduced markers of inflammation. Cinaciguat also protected vasculature from oxidative stress and improved vascular responsiveness. In canine coronary arteries exposed to the oxidant peroxynitrite, cinaciguat improved endothelium-dependent relaxation and reduced inflammation, as indicated by decreased nitrotyrosine protein residues [99]. The timing of sGC stimulation or activation is important. For example, treatment with a single oral dose of cinaciguat during ischemia did not protect against cardiac IR [107]. In mice subjected to coronary artery occlusion for 30 min, however, intraperitoneal cinaciguat administered prior to ischemia reduced myocardial injury in wild type but not cardiomyocyte-specific sGC knockout mice [95]. sGC activation is more beneficial prior to, as opposed to during, cardiac IR [96]. Riociguat, another heme-dependent sGC stimulator, also reduces myocardial IR injury [97]. Following coronary artery ligation, mice administered riociguat had smaller infarcts and had preserved left ventricular systolic function compared to a control. This preservation persisted at 28 days after treatment, indicating a lasting effect of sGC treatment on IR injury and subsequent cardiac function. Other work corroborates the findings that both sGC stimulators and activators have beneficial effects on myocardial infarct size, though simultaneous administration of these drugs does not improve their efficacy [82].

There are several possible mechanisms underlying these benefits. One such mechanism may be improvements in cardiac microcirculation during reperfusion [98]. In a canine model of cardiopulmonary bypass (CPB) with cardioplegic arrest, sGC activation with intravenous cinaciguat prior to CPB increased coronary blood flow and improved biventricular contractility post CPB [99]. These findings were associated with increased myocardial adenosine triphosphate content and improved endothelium-dependent vasodilation in peroxynitrite-injured coronary vessels. sGC stimulators and activators also reduce mitochondrial superoxide production, reduce endothelial intercellular gap formation, and reduce myocardial edema in reperfused hearts [100,101]. These cardioprotective effects are mediated by cGMP-induced PKG activation [100]. One study found that eNOS inhibition with L-NAME slightly decreased sGC stimulator-induced protection, suggesting that endogenous NO enhances these effects but is not required [82]. In this study, the addition of an sGC stimulator increased cGMP concentrations, but this did not increase left ventricular protection. While L-NAME treatment did not eliminate the protective effect of sGC stimulation, it did eliminate the rise in cGMP after stimulation. Together, these findings suggest that the presence of sGC is essential for stimulators and activators to induce their protective effects, but these effects are mediated by more than the increase in cGMP concentration. Additional research is necessary to determine what additional mechanisms may contribute to these protective effects.

### 5.2. Cerebral Protection

Cerebral protection from IR injury is another application of sGC potentiation with promising potential. In a mouse model of transient middle cerebral artery occlusion, for example, sGC alpha subunit levels were markedly decreased in brain tissue following stroke, and sGC was poorly responsive to NO [102]. In this model, heme-free sGC from post-ischemic brain tissue had increased responsiveness to an sGC activator compared to control tissue. Subsequently, post-stroke treatment with a hemodynamically insignificant dose of an sGC activator decreased cerebral infarct volume in transient MCA occlusion. sGC activator treatment also reduced blood–brain barrier disruption and increased post-stroke cerebral blood flow without affecting systemic blood pressure. These differences suggest that a possible mechanism for the improvement in IR injury is related to the effects of sGC activation on the affected vasculature.

sGC stimulation reduces cerebral inflammation in animal models. The application of the sGC stimulator CYR119 decreased the expression of inflammatory genes and cytokines in a rat model of in vivo lipopolysaccharide and diet-induced neuroinflammation, potentially related to reduced BBB disruption and leukocyte infiltration [103,108]. These findings are in line with prior data suggesting that the NO-sGC-cGMP pathway modulates neuroinflammation through the activation of protein kinase G and that augmentation of this pathway with NO-donors or phosphodiesterase inhibitors decreases the expression of leukocyte adhesion molecules and promotes the resolution of inflammation [109,110,111].

### 5.3. Renal, Pulmonary, and Vascular Protection

sGC agonists have also demonstrated protection in other tissues and organs. For example, the sGC stimulator riociguat reduced systemic hypertension, improved systolic heart function, and increased overall survival in rats fed high sodium diets to model pressure and volume overload of the heart [93]. sGC stimulation furthermore significantly reduced fibrotic tissue remodeling in both the renal cortex and myocardium. Similar protective effects have been observed in models of chronic kidney disease. In proliferative glomerulonephritis, sGC stimulation reduced mesangial proliferation, extracellular matrix accumulation, and proteinuria [112]. In a model of obstructive renal disease, sGC stimulation during unilateral ureteral obstruction increased cGMP production and decreased tubulointerstitial fibrosis and inflammation, as well as tubular atrophy and apoptosis [86]. As previously discussed, IR injury may result in alterations in leukocyte adhesion via the upregulation of leukocyte–endothelium adhesion, and sGC stimulation reduced these interactions, endothelial activation, and organ dysfunction in sickle-cell-disease-related kidney injury [108]. Notably, sGC stimulation reduced kidney injury in these settings regardless of the underlying etiology of the renal damage. Riociguat treatment reduces both renal and cardiac damage in both low- and high-renin models of hypertensive disease, and vascular relaxation to riociguat was preserved in a nitrate tolerance model [113]. Therefore, vascular effects may mediate these renal observations. In a murine study of variable oxygen tension (PaO_2_ range 104–318 mmHg) during renal IR injury, aortic responses to endothelium-dependent and -independent vasodilation were impaired in hyperoxia treated animals, while the response to the sGC activator cinaciguat was unaffected by oxygen tension [62].

These benefits of sGC modulation are not limited to renal tissue. Recent work with the sGC stimulator praliciguat improved perfusion after limb ischemia in a model of peripheral arterial disease and reduced fibrosis in models of non-alcoholic steatohepatitis [104,105]. In an isolated lung model of pulmonary IR, sGC stimulation with BAY 41-2272 attenuated the degree of lung injury. It both decreased pulmonary arterial pressure during reperfusion and decreased ROS production, a major contributor to lung IR injury [106].

These studies suggest that the protective effects of sGC stimulation and activation are broad and that sGC effects on vasculature during IR may underlie benefits of sGC stimulation and activation across different organs.

### 5.4. Soluble Guanylyl Cyclase Stimulation in Humans and Future Potential Uses

The extensive pre-clinical evidence demonstrating potential benefits of sGC stimulators and activators has led to clinical trials to treat acute and chronic diseases. Some benefits of sGC augmentation have been observed as well as some negative side effects. The clinical studies of sGC stimulators and activators are summarized in Table 2.

Despite the promising evidence in pre-clinical models, the sGC activator cinaciguat has been poorly tolerated in humans. An uncontrolled study of patients with acute heart failure demonstrated that cinaciguat reduced cardiac preload and afterload and increased cardiac output [119]. Renal function was preserved, but 13 of the 60 patients in this study had adverse effects, most commonly hypotension. In subsequent trials, cinaciguat treatment was limited by symptomatic non-fatal hypotension. In the COMPOSE trial, for example, patients hospitalized with heart failure were treated with placebo or fixed-dose intravenous cinaciguat infusion [120]. Cinaciguat increased hypotension with no improvement in dyspnea, cardiac index, or renal function. This trial was stopped early. In a subsequent trial that titrated cinaciguat to systolic blood pressure, heart rate, and participant tolerability, moderate and high dose cinaciguat induced hypotensive events, and the trial was again stopped early for safety concerns [121]. In this study, cinaciguat-treated patients had higher troponin I levels and greater incidence of ventricular tachycardia than placebo-treated patients. The results of these trials have limited further study of cinaciguat due to its unfavorable pharmacodynamic and side effect profiles. Cinaciguat has not been approved for clinical use.

The sGC stimulator riociguat has been more successful in clinical trials. In patients with pulmonary hypertension, riociguat decreased mean pulmonary arterial pressure, pulmonary vascular resistance, and cardiac index in a dose-dependent manner and to a greater extent than inhaled NO, without increased adverse events [114]. Symptomatic improvements in dyspnea and functional class were also observed in participants treated with riociguat [124]. These findings were consistent across pulmonary hypertension etiologies, including primary pulmonary arterial hypertension (PAH), chronic thromboembolic pulmonary hypertension (CTEPH), and pulmonary hypertension due to interstitial lung disease (PH-ILD) [92,114,124,125,126]. Riociguat was FDA-approved for use in both PAH and CTEPH in 2013. In phase III clinical trials, riociguat led to significant improvements in exercise capacity, pulmonary vascular resistance (PVR), and World Health Organization functional class in both PAH and CTEPH, while dyspnea score and time to clinical worsening were improved in PAH alone [8,115,116]. Riociguat has, furthermore, been studied for the treatment of heart failure. In patients with heart failure, it improved cardiac index, stroke volume index, and PVR but did not improve clinically important outcomes such as exercise tolerance or functional status [117,118,127]. More recently developed sGC stimulators have been found to be more effective in the treatment of heart failure.

The sGC stimulator vericiguat improves outcomes in patients with heart failure. Initial data suggested that it was well tolerated by patients, while anemia and clinically insignificant decreases in arterial pressure were the most common side effects [128]. In the phase III VICTORIA trial, over 5000 patients with heart failure with reduced ejection fraction (HFrEF), defined as an left ventricular ejection fraction < 45%, were treated with daily oral vericiguat or placebo [122]. Vericiguat reduced the composite endpoint of all-cause mortality or hospitalization for heart failure with a hazard ratio of 0.90 (95% CI: 0.83 to 0.98). Importantly, symptomatic hypotension was not significantly different between the vericiguat group and the placebo group. As a result of this trial, vericiguat was approved by the FDA for the treatment of heart failure with reduced ejection fraction in 2021. Vericiguat did not improve functional capacity in patients with heart failure and preserved ejection fraction compared to placebo in the phase IIb VITALITY-HFpEF clinical trial, therefore its clinical use to date is limited to patients with HFrEF [123].

The specific mechanism underlying improvement in outcomes among HFrEF patients is not known, although in early phase trials vericiguat increased cardiac output and decreased systemic vascular resistance compared to placebo. There remain many more potential clinical uses for sGC activators and stimulators. sGC stimulators have demonstrated better safety profiles than activators and have demonstrated clinical improvement in patients, perhaps related to decreased pulmonary and systemic vascular resistance and increased cardiac performance. The clinical utility of sGC activators and stimulators during IR requires additional investigation.

## 6. Conclusions and Future Directions

sGC stimulation and activation are promising potential treatments for IR injuries. Extensive pre-clinical findings support the benefit of sGC stimulation and activation in IR-related heart, brain, lung, kidney, liver, and vascular injury. These protective effects may result from an improved regulation of blood flow, decreased oxidative injury, and/or decreased inflammation. While clinical trials have noted a benefit of the sGC stimulators riociguat and vericiguat on the treatment of pulmonary hypertension and heart failure with reduced ejection fraction, there is a relative paucity of clinical investigations focused on the effects of sGC stimulation and sGC activation on clinically significant IR injury. Additional investigation into the protective effects of sGC stimulation and activation in IR settings in human patients is warranted.

## Figures and Tables

**Table 1:** Pre-clinical studies of sGC stimulators and activators during IR injury.

Citation	Drug	Model	Findings
Korkmaz et al. [94]	Cinaciguat	Global cardiac ischemia	Cinaciguat reduced oxidative stress, reduced inflammatory marker expression, improved histopathological lesions, and improved cardiac performance.
Korkmaz et al. [94]	Cinaciguat	Coronary oxidation injury	Cinaciguat improved endothelium-dependent relaxation and reduced vascular inflammation
Frankenreiter et al. [95]	Cinaciguat	Cardiac IR	Intraperitoneal cinaciguat prior to LAD clamping reduced cardiac injury in sGC WT mice but not sGC KO mice.
Salloum et al. [96]	Cinaciguat	Cardiac IR	sGC treatment prior to ischemia reduced infarct size more than sGC treatment during reperfusion. Cinaciguat increased PKG activity and H_2_S generation.
Methner et al. [97]	Riociguat	Cardiac IR	Riociguat reduced infarct size after LAD ligation and improved LV systolic function preservation. Effects persisted at 28 days after injury.
Bice et al. [82]	BAY 41-2272, BAY 60-2770	Cardiac IR	sGC stimulators and activators reduced infarct size in rat hearts after reversible LCA occlusion, but simultaneous treatment with stimulators and activators did not further improve efficacy. eNOS inhibition attenuated the protective effect of sGC stimulation. cGMP levels did not correlate with the degree of LV protection. eNOS inhibition eliminated the rise in cGMP after sGC stimulation but did not eliminate its protective effect.
Cai et al. [98]	Vericiguat	Cardiac IR	sGC stimulation reduced myocardial IR injury by improving coronary microcirculation.
Radovitz et al. [99]	Cinaciguat	Cardiac IR	Pre-incubation of coronary rings with cinaciguat reduced peroxynitrite-induced endothelial dysfunction and restored vasodilatory responses to acetylcholine. Cinaciguat infusion prior to CPB increased LV and RV contractility recovery and increased coronary blood flow.
Lee et al. [100]	BAY 60-2770	Cardiac IR	sGC activation decreased mitochondrial superoxide production and decreased myocardial injury via cGMP-activation of PKG.
Kasseckert et al. [101]	HMR1766	Cardiac IR	sGC activation reduced endothelial intercellular gap formation and reduced myocardial edema.
Langhauser et al. [102]	BAY 60-2770, BAY 58-2667	Cerebral IR	sGC activator treatment reduced cerebral infarct volume at 24 h in transient but not permanent MCA occlusion. It also reduced BBB leakage and increased post-stroke cerebral blood flow without affecting systemic BP.
Correia et al. [103]	CYR119	Neuro-inflammation	sGC stimulator treatment reduced inflammatory gene expression and cytokine production in LPS and diet-induced neuroinflammation.
Geschka et al. [93]	Riociguat	Hypertension	sGC stimulation prevented renal fibrotic tissue remodeling in systemic hypertension.
Hall et al. [104]	Praliciguat	Cirrhosis	sGC stimulation inhibited hepatic fibrosis and inflammation in NASH cirrhosis.
Foussard et al. [105]	Praliciguat	Limb ischemia	sGC stimulation improved perfusion and foot function as well as increased arteriole diameter and reduced ICAM-1 expression in ischemic limb injuries.
Egemnazarov et al. [106]	BAY 41-2272	Pulmonary IR	sGC stimulation reduced IR-induced lung injury by decreasing vascular permeability and ROS production.
Mace et al. [62]	Cinaciguat	Renal IR	Hyperoxic conditions increased superoxide levels and impaired endothelium-dependent and -independent relaxation, while sGC activation was unaffected.

BBB, blood brain barrier; BP, blood pressure; cGMP, cyclic guanosine monophosphate; CPB, cardiopulmonary bypass; eNOS, endothelial nitric oxide synthase; ICAM-1, intracellular adhesion molecule-1; IR, ischemia-reperfusion; KO, knockout; LAD, left anterior descending; LCA, left coronary artery; LV, left ventricle; MCA, middle cerebral artery; NASH, non-alcoholic steatohepatitis; PKG, protein kinase G; ROS, reactive oxygen species; RV, right ventricle; sGC, soluble guanylyl cyclase; WT, wild type.

**Table 2:** Clinical studies of sGC stimulators and activators.

Citation	Drug	Model	Findings
Grimminger et al. [114]	Riociguat	PH	Riociguat reduced PVR and improved PAP and cardiac index to a greater extent than inhaled NO and was well tolerated.
Ghofrani et al. [115]	Riociguat	CTEPH	Riociguat significantly improved exercise capacity as well as PVR, NT-proBNP level, and WHO functional class.
Ghofrani et al. [116]	Riociguat	PAH	Riociguat significantly improved exercise capacity as well as PVR, NT-proBNP levels, WHO functional class, time to clinical worsening, and Borg dyspnea score.
Bonderman et al. [117]	Riociguat	PH caused by LV systolic dysfunction	Riociguat did not affect the primary endpoint, mPAP, but it increased cardiac index and stroke volume index and decreased PVR and SVR. Riociguat improved health-related quality of life but did not affect clinical worsening events and change in functional class.
Hoeper et al. [118]	Riociguat	PH due to ILD	Riociguat improved cardiac output and pulmonary vascular resistance but not mPAP, 6 min walk duration, functional class, and quality of life measures.
Lapp et al. [119]	Cinaciguat	Acute decompensated heart failure	Cinaciguat reduced cardiac preload and afterload and increased cardiac output while preserving renal function.
Gheorghiade et al. [120]	Cinaciguat	Heart failure	Constant-dose cinaciguat infusions caused hypotension without improving dyspnea, cardiac index, or renal function. Trial stopped early.
Erdmann et al. [121]	Cinaciguat	Heart failure	Titratable-dose cinaciguat infusions caused hypotension at moderate and high doses. Trial stopped early.
Armstrong et al. [122]	Vericiguat	HFrEF	Vericiguat reduced the composite endpoint of all-cause mortality or hospitalization for heart failure without causing symptomatic hypotension.
Armstrong et al. [123]	Vericiguat	HFpEF	Vericiguat did not improve the physical limitation score compared to placebo after 24 weeks of treatment for patients with recent decompensation.

CTEPH, chronic thromboembolic pulmonary hypertension; HFpEF, heart failure with preserved ejection fraction; HFrEF, heart failure with reduced ejection fraction; ILD, interstitial lung disease; LV, left ventricle; NT-proBNP, N-terminal-pro-brain natriuretic peptide; PAH, pulmonary arterial hypertension; PAP, pulmonary artery pressure; PH, pulmonary hypertension; PVR, pulmonary vascular resistance; SVR, systemic vascular resistance; WHO, World Health Organization.

## Data Availability

Not applicable.
